# Arteriovenous malformation with pseudoaneurysm on the left upper limb

**DOI:** 10.1002/ccr3.6026

**Published:** 2022-07-11

**Authors:** Akihisa Furuta, Daisuke Futagami, Hironobu Morimoto, Junya Kitaura, Shogo Mukai

**Affiliations:** ^1^ Department of Cardiovascular Surgery Fukuyama Cardiovascular Hospital Hiroshima Japan

**Keywords:** arteriovenous malformation, Pseudoaneurysm

## Abstract

A 61‐year‐old woman developed a pulsatile mass on the left upper limb and was diagnosed with arteriovenous malformation with pseudoaneurysm. A two‐stage operation including ligation and resection of the aberrant branches and subsequent resection of the mass with revascularization was performed. Histological analysis suggested arteriovenous malformation and pseudoaneurysm.

## Graphical abstract

Ligation and resection of the aberrant branches and resection of the mass with revascularization relieved clinical symptoms. Long‐term follow‐up is required for monitoring a progression of a new arteriovenous network and aneurysm..

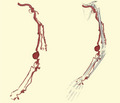

## 1 INTRODUCTION

An arteriovenous malformation (AVM) is a vascular system anomaly that causes an arteriovenous shunt, which can lead to cardiac failure in some cases. The majority of AVMs are found in people between the ages of 20 and 40 years.^1^ Although an AVM can appear anywhere in the body, more than 50% of all AVMs are found in the brain or spine and AVMs in the extremities are uncommon. Because of the rarity of AVMs, information regarding the occurrence of AVMs in the extremities with appropriate histological findings is scarce. Herein, we report a case of AVMs accompanied by a pseudoaneurysm with pathological considerations.

## 2 CASE REPORT

**FIGURE 1:**
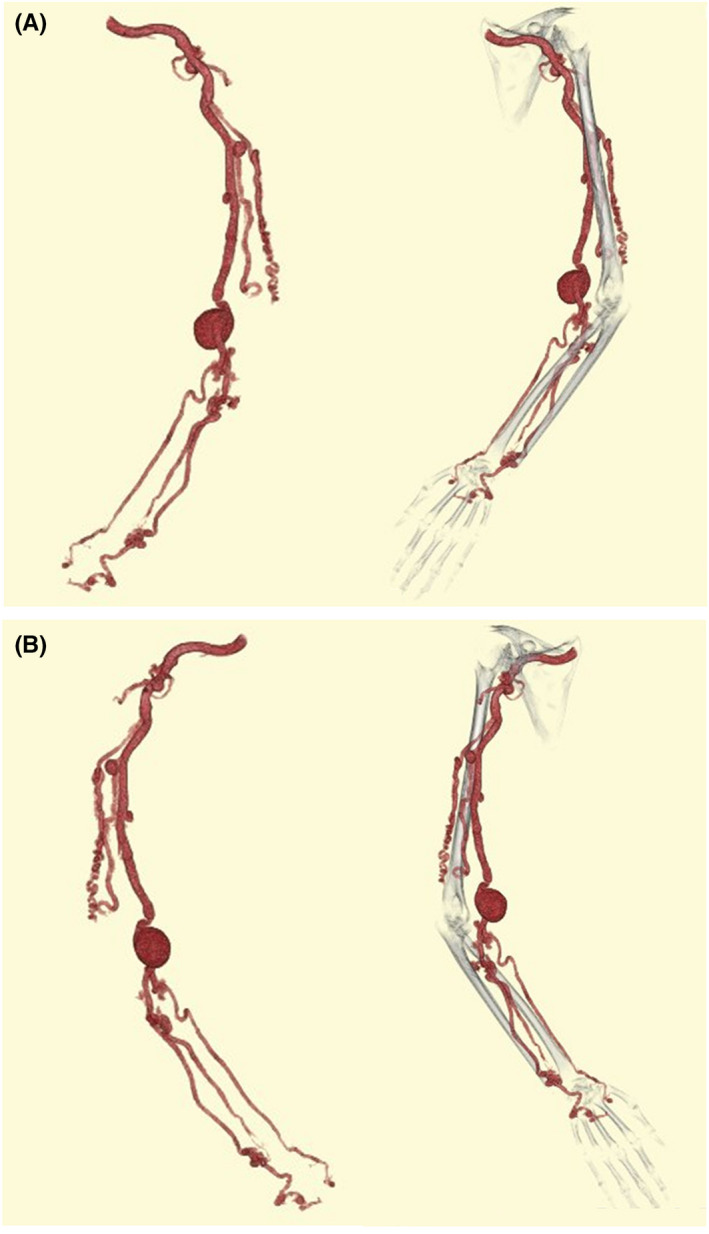
Computed tomography of the left upper limb. (A) Front (B) Back. An 8 mm dilated left brachial artery with several aberrant branches and small aneurysms. A 3 × 3 cm pseudoaneurysm was also seen before the bifurcation of the radial artery. Beyond the pseudoaneurysm, arteriovenous malformations and small aneurysms were found in the forearm

A 61‐year‐old woman with no history of trauma or hemodialysis was referred to our hospital for treatment after developing a pulsing mass on her left upper limb. The swelling was first noticed in early childhood, with a significant size disparity between her two upper limbs; however, it was left untreated. Physical examination revealed skin pigmentation, varicosities, a palpable thrill, and continuous bruit on the left upper limb, as well as a significant size discrepancy between the two upper limbs. A blood test and echocardiogram revealed no signs of congestive heart failure. Computed tomography revealed an 8‐mm dilated brachial artery with several abnormal branches, a 3 × 3‐cm pseudoaneurysm, and AVMs on the forearm (Figure 1A,B). Angiography also revealed that the first branch of the brachial artery networked AVMs and several other abnormal arteries branching off from the brachial artery (Figure S1).

There was a need to resect surgically the mass due to the risk of a rupture. However, a single operation of resecting the mass was considered to be associated with a risk of operative bleeding because a blood pressure of all veins was equivalent to that of an artery and a swelling of the left upper limb could lead to a surgical wound site‐related complication such as dehiscence and infection. Therefore, AVMs should have been treated to reduce the vein pressure and alleviate the swelling. As all AVMs cannot be completely removed and a large AVM cannot be treated with catheter intervention, a surgical treatment seemed to be an optimal option. Then, we decided to perform a two‐stage surgery including initial resection of the aberrant branches of the brachial artery and subsequent resection of the pseudoaneurysm.

**FIGURE 2:**
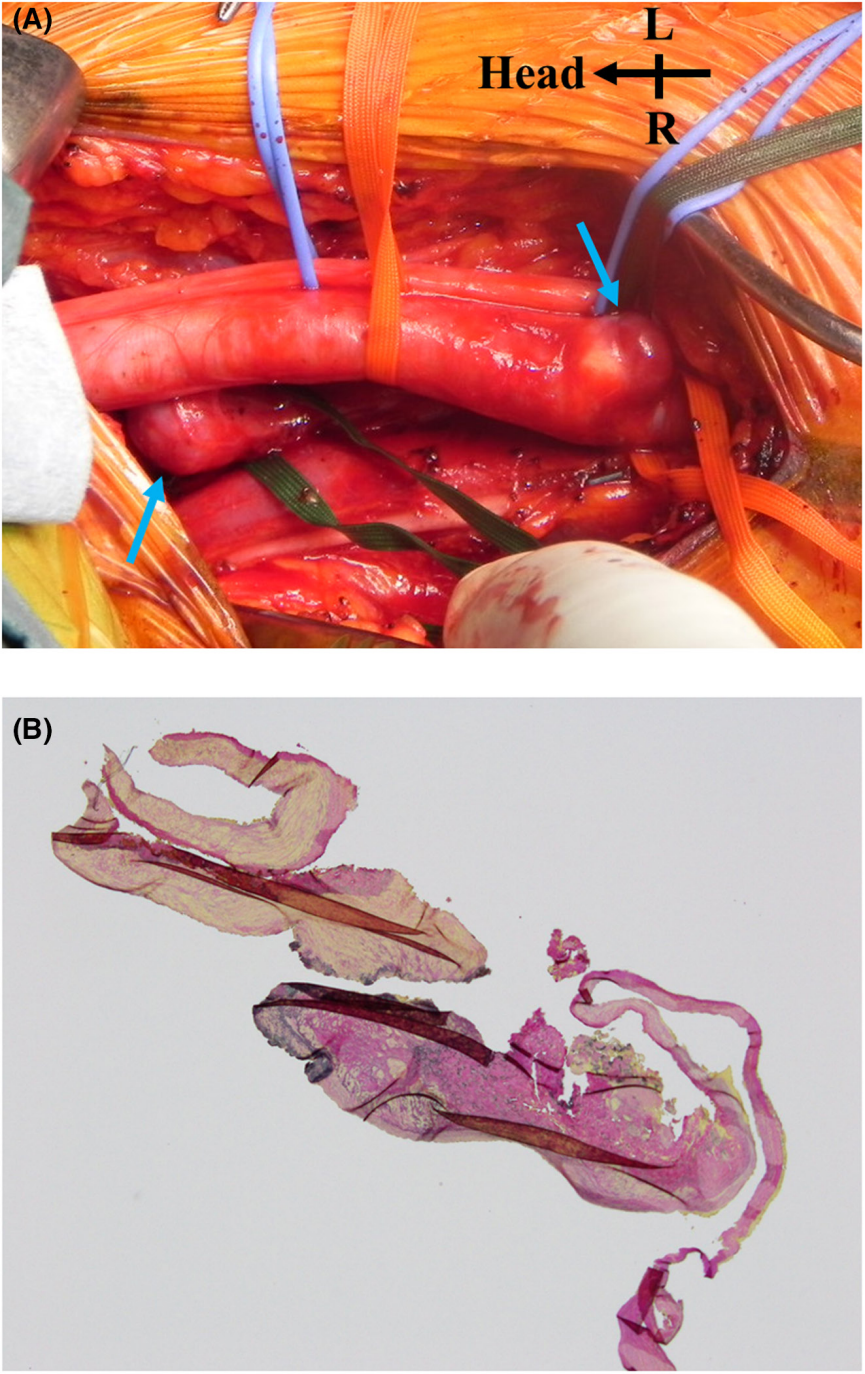
First operation. (A) Operative findings. A thin, dilated, brachial artery with the aberrant branched vessel and a small aneurysm (arrow). (B) Elastica van Gieson stain of the abnormal branch, magnification ×4. The aberrant vessels were composed of collagen fibers and smooth muscle that lack elastic fibers, suggesting arteriovenous malformation

In the first surgery, three aberrant vessels branching from the middle of the brachial artery were ligated and resected under general anesthesia (Figure 2A). The swelling and continuous bruit on her left upper limb improved, and she was discharged without any incident on the fifth postoperative day. Histological analysis revealed that the aberrant vessels lacked elastic fibers, and the tissue was composed of collagen fibers and smooth muscle, suggesting that they were AVMs (Figure 2B).

**FIGURE 3:**
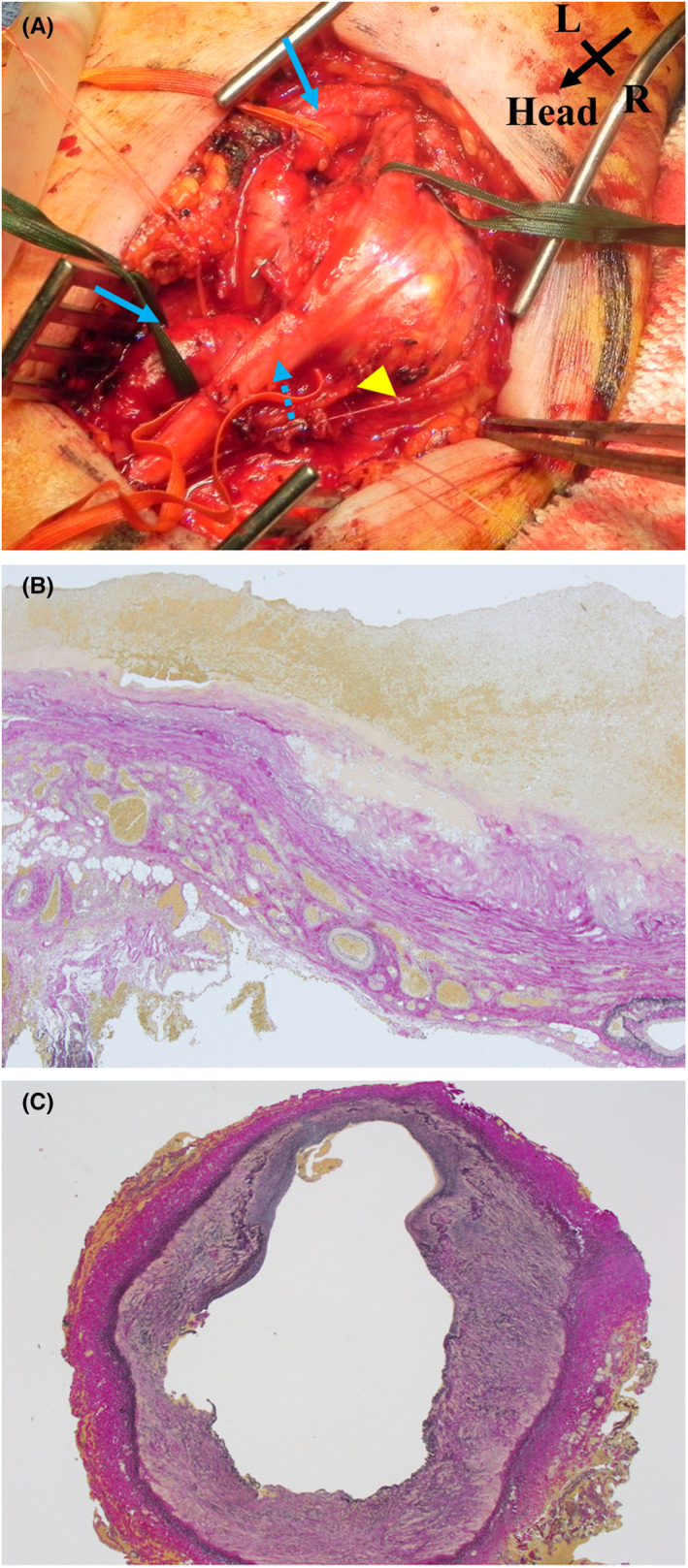
Second operation. (A) Operative findings. The brachial artery (arrow) is thin, dilated, and twisted around the mass. The mass is 3 × 3 cm (triangle), involving the median nerve (dotted arrow). The aberrant branch of the brachial artery was ligated and resected. After resection of the mass, revascularization of the brachial artery was performed via direct anastomosis. (B) Elastica van Gieson stain of the mass, magnification ×10. The wall of the mass lacked a vascular structure accompanied by elastic lamina and was composed of fibrillar connective tissues with tiny vessels, which is a characteristic histological picture of a pseudoaneurysm. (C) Elastica van Gieson stain of the brachial artery, magnification ×4. The wall of the brachial artery was composed of a fragmented internal elastic layer and fibrillated tunica media of the brachial artery, which had histological properties comparable to a vein

The second surgery was performed under general anesthesia 1 month after the initial surgery. The median nerve was encircled by a 3 × 3 cm mass that was a portion of the thin, dilated brachial artery (Figure 3A). The aberrant vessel that branched off the brachial artery was ligated and removed. The mass was removed after clamping the brachial artery, and the brachial artery was revascularized via direct anastomosis. The patient was discharged on the seventh postoperative day without any incident. According to the Elastica‐van Gieson histological analysis, the mass's wall lacked elastic lamina and was composed of fibrillar connective tissues, including tiny vessels, which is a characteristic histological picture of a pseudoaneurysm (Figure 3B). The wall of the brachial artery was composed of a fragmented internal elastic layer and a fibrillated tunica media, which was similar to the vein structure (Figure 3C).

One year after the operation, the skin pigmentation, palpable thrill, and significant size discrepancy between the two upper limbs disappeared, and the varicosities were alleviated.

## 3 DISCUSSION

We believe the AVM, in this case, was congenital because the patient exhibited symptoms in the left upper limb since childhood. The vascular system develops in four stages: blood islands, vasculogenesis, angiogenesis, and maturation.^2^ The developmental stage can halt at any time during vessel construction, resulting in a variety of vascular abnormalities. A failure of the angiogenesis phase, on which the identification of artery and vein hinges, may be involved in an AVM.^3^ Because of the collagen fiber and smooth muscle with a lack of elastic fiber in the first surgery, our pathology study suggested that aberrant arteries were AVMs, and the structure of the brachial artery was comparable in histological features to a vein in the second operation. According to these findings, AVM development failure may occur before angiogenesis. Pathological analysis of the mass revealed that it was a pseudoaneurysm because of the lack of elastic lamina and the presence of fibrillar connective tissues, including tiny capillaries. AVM vessels are thin and fragile, and as a result of excessive blood flow and circulatory deficit of the vasa vasorum, they may suffer degenerative changes, resulting in a collapsed structure such as a pseudoaneurysm.

Therapeutic options for AVMs include endovascular, surgical, radiation therapy, or a combination of all of these. However, treatment for AVM is challenging because clinical conditions vary from one case to another, and, additionally, a large widespread AVM cannot be completely repaired.^4^ In our case, where several aberrant arteries that branched from the brachial artery provided the connection with the vein, as endovascular treatment may not exclude the connection between arteries and veins, we thought surgical resection of the abnormal arteries involving AVM and the mass would lead to abating symptoms and long‐term success. We performed a two‐stage operation that resulted in the improvement of several of the symptoms. However, because other aberrant arteries linking the vein remain and there are worries regarding the progression of a new arteriovenous network and aneurysm, long‐term follow‐up should be performed.

## 4 CONCLUSION

The patient's symptoms were relieved after the aberrant arteries that formed the AVM and pseudoaneurysm were successfully resected and revascularized. Long‐term monitoring will be necessary since other aberrant arteries linking the vein still exist, and there are concerns about the progression of a new arteriovenous network and aneurysm.

## AUTHOR CONTRIBUTIONS

Akihisa Furuta conceptualized and designed the study, and drafted, critically revised and approved the article. Daisuke Futagami conceptualized and designed the study, interpreted the data, and approved the article. Hironobu Morimoto conceptualized and designed the study and critically revised the article. Junya Kitaura and Shogo Mukai critically revised and approved the article.

## CONFLICT OF INTEREST

None.

## ETHICAL APPROVAL

Approval of the International Review Board was not required at our institution because this study was a case report.

## CONSENT

Written informed consent was obtained from the patient for the publication of this report under the journal's patient consent policy.

## Supporting information


Figure S1

## Data Availability

The data that support the findings of this study are available from the corresponding author upon reasonable request.
