# Supplementary figures and images for: Arteriovenous malformation with pseudoaneurysm on the left upper limb
Source: Clin Case Rep. 2022 Jul 11;10(7):e6026. doi: 10.1002/ccr3.6026 (PMC9272232; doi:10.1002/ccr3.6026)

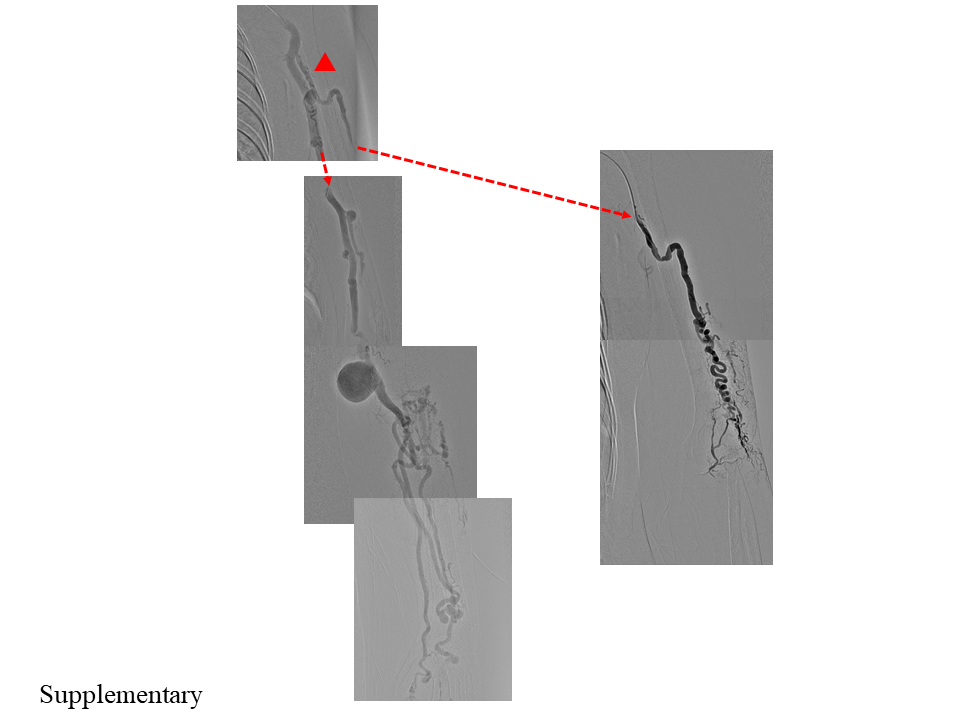

Supplement: Supplementary file 1 — Figure S1 [file CCR3-10-e6026-s001.TIF]
